# Sex-Related Safety Signals of Sotorasib in Non-Small Cell Lung Cancer: A Real-World, Pharmacovigilance Study from the EudraVigilance Database

**DOI:** 10.3390/ph18101574

**Published:** 2025-10-19

**Authors:** Desirèe Speranza, Mariapia Marafioti, Martina Musarra, Vincenzo Cianci, Fausto Omero, Calogera Claudia Spagnolo, Marco Calabrò, Nicola Silvestris, Natasha Irrera, Mariacarmela Santarpia

**Affiliations:** 1 Department of Chemical, Biological, Pharmaceutical and Environmental Sciences, University of Messina, 98166 Messina, Italy; desiree.speranza@gmail.com; 2 Department of Clinical and Experimental Medicine, University of Messina, 98122 Messina, Italy; natasha.irrera@unime.it; 3 Medical Oncology Unit, Department of Human Pathology “G.Barresi”, University of Messina, 98125 Messina, Italy; marafiotimariapia@gmail.com (M.M.); martimusa.mm@gmail.com (M.M.); faustoomero@hotmail.it (F.O.); 4 Department of Biomedical and Dental Sciences and Morphofunctional Imaging, University of Messina, 98125 Messina, Italy; enzocianci.1997@gmail.com (V.C.); spagnoloclaudia92@gmail.com (C.C.S.);; 5 Medical Oncology Unit, IRCCS Istituto Tumori “Giovanni Paolo II”, 70124 Bari, Italy; n.silvestris@oncologico.bari.it

**Keywords:** sotorasib, KRAS G12C, NSCLC, pharmacovigilance, EudraVigilance studies, sex-related differences

## Abstract

**Background**: Sotorasib, a KRAS G12C inhibitor, is approved for treating non-small cell lung cancer (NSCLC) and has shown a distinct safety profile in randomized clinical trials (RCTs). However, post-marketing pharmacovigilance is crucial to identify real-world safety signals including sex-specific differences that may not be evident in controlled trial settings. **Methods**: This analysis reviewed 845 individual case safety reports (ICSRs) from the EudraVigilance (EV) database between 1 January 2021, and 8 April 2025, involving NSCLC patients treated with sotorasib. Adverse drug reactions (ADRs) were assessed by sex, seriousness, outcome, and system organ class (SOC). Disproportionality analyses were conducted to detect sex-specific safety signals, and results were compared with data from the CodeBreaK200 RCT by using a two-proportion z-test. **Results**: Among the ICSRs, 49.2% involved male and 40.1% female patients. Serious ADRs accounted for 47.5% of cases, with females at higher risk (relative risk [RR] = 1.31; 95% confidence interval (CI): 1.22–1.40; *p* < 0.0001). The most frequently reported SOCs were neoplasms (15.8%), gastrointestinal disorders (15.3%), and hepatobiliary disorders (11.5%). Four sex-specific safety signals were identified: women had a significantly increased risk of cholestasis (RR = 3.37) and hepatotoxicity (RR = 3.01), while men were less likely to report decreased appetite (RR = 0.20) and rash (RR = 0.14). Real-world data showed lower reporting of diarrhea, fatigue, nausea, and liver enzyme elevations (*p* < 0.0001). **Conclusions**: Real-world pharmacovigilance supports the RCT findings and highlights sex-specific risks, thus emphasizing the importance of sex-aware monitoring and personalized toxicity management.

## 1. Introduction

Non-small cell lung cancer (NSCLC) is the second most frequently diagnosed cancer and the leading cause of cancer-related deaths worldwide, accounting for an estimated 2.48 million new cases and 1.82 million deaths in 2022. Despite significant advances in diagnostics and therapeutics, the 5-year net survival rate remains poor—typically below 20% in most countries [1]. NSCLCs are classified into non-oncogene-addicted and oncogene-addicted subtypes, based on the absence or presence of specific genetic alterations that drive tumorigenesis [2,3]. Molecular profiling has revolutionized the clinical management of NSCLC, improving diagnosis, prognostication, and enabling personalized treatment strategies. The adoption of next-generation sequencing (NGS) techniques has facilitated the detection of key driver genomic alterations—including EGFR, BRAF, KRAS, HER2 and MET mutations and ALK, ROS1, RET, NTRK rearrangements—critical for selecting targeted therapies [4].

KRAS activating mutations represent the most prevalent oncogenic drivers in lung adenocarcinoma, occurring in approximately 25% to 32% of cases across both early and advanced stages of the disease [5], whereas these mutations are detected in less than 1% of squamous cell carcinoma cases [6].

In NSCLC, KRAS mutations are predominantly single-base missense mutations that cluster in three hotspots—codons G12, G13, and Q61—mainly located in exons 2 and 3. Among all KRAS mutations in NSCLC, the most prevalent is the single-nucleotide variant p.G12C, which results in a glycine-to-cysteine substitution at codon 12 in exon 2 and accounts for approximately 40% of KRAS mutations, with an overall prevalence of about 13% in lung adenocarcinoma [7,8,9,10].

A range of alterations is observed in non-G12C KRAS-mutant NSCLC; these variations include other point mutations at codon 12 of exon 2 such as KRAS G12V and G12D, which account for approximately 21% and 17% of KRAS mutations, respectively [11]. Other variants include KRAS G12A, G12S, G12R, and G12F. Point mutations in exon 2 codon 13, such as KRAS G13S as well as KRAS gene amplifications, have also been reported. In addition, mutations in exon 3 codon 61, including Q61K, Q61A, Q61H, and Q61L, account for approximately 35–40%, 40–45%, 5–7%, and 1% of all non-G12C KRAS mutations, respectively [6,12,13,14,15].

Co-occurring KRAS mutations have been identified in around 3.4% of KRAS-mutated tumors, with a higher prevalence (8%) in KRAS G12C-mutant cases. Co-mutations in other genes are common and clinically relevant and may significantly influence tumor biology, prognosis, and therapeutic response [16]. In particular, G12F and G12V represent the most frequently observed concurrent KRAS mutations [17]. TP53 mutation was also identified in ~40% of cases as well as serine/threonine kinase 11 (STK11) and Kelch-like ECH-associated protein 1 (KEAP1) mutations in ~20% and 13–24% of cases, respectively [10]. Moreover, a rare co-occurrence of targetable mutations in EGFR (1.2%) and BRAF (1.3) was noted. Specific co-mutation patterns were associated with distinct KRAS mutation subtypes. Notably, KRAS G12C mutations were linked to ERBB2 amplifications, while G12V and G13X variants frequently co-occurred with mutations in the phosphatase and tensin homolog (PTEN) gene. In contrast, patients harboring G12D mutations showed a high prevalence of concurrent platelet-derived growth factor receptor alpha (PDGFRA) mutations and a lack of EGFR alterations [9].

KRAS mutations are also associated with elevated programmed death-ligand 1 (PD-L1) expression, contributing to immune evasion through T-cell exhaustion mechanisms [18]. The prognostic implications of KRAS mutations have been widely documented. Particularly, patients with the G12C variant tend to have worse clinical outcomes than those with non-G12C KRAS mutations or KRAS wild-type tumors [19]. Furthermore, these patients exhibit a higher frequency of metastases at diagnosis (94% vs. 88%) [20].

Although KRAS mutations are highly prevalent and play a pivotal role in tumor growth and survival, KRAS was long regarded as “undruggable” due to its high affinity for guanosine triphosphate (GTP) and the absence of accessible binding pockets. Recently, the development of covalent inhibitors specifically targeting KRAS G12C, such as sotorasib (AMG510) and adagrasib (MRTX849), has overturned this paradigm. These drugs irreversibly bind to the cysteine residue at position 12 within the switch II pocket of KRAS when the protein is in its inactive GDP-bound conformation, effectively locking it in this state [21]. As a result, the ability of KRAS to propagate oncogenic signaling is inhibited, leading to reduced cellular proliferation and the induction of tumor cell apoptosis [22]. Notably, sotorasib exhibits high selectivity for the G12C mutant form of KRAS, thereby sparing wild-type KRAS and reducing off-target toxicity. The CodeBreaK 100 clinical trial demonstrated the clinical benefit of sotorasib in patients with advanced NSCLC harboring KRAS G12C mutations. This led to Food and Drug Administration (FDA) approval for patients who had received at least one prior line of systemic therapy [23].

In contrast, patients with non-G12C KRAS mutations currently receive the same treatment approaches as KRAS wild-type patients due to the absence of approved targeted therapies for these KRAS variants. While molecular predictors of sotorasib efficacy have been described, the identification of features associated with a higher risk of toxicity still represents a clinical challenge [24]. In this context, in the present study, we aimed to describe the safety profile of sotorasib and identify clinical characteristics that could be useful to predict toxicity in a large real-world database.

## 2. Results

### 2.1. General Characteristics in the Real-World Population

From 1 January 2021 to 8 April 2025, a total of 1477 ICSRs were collected, out of which 845 ICSRs were included in the analysis. The excluded ICSRs pertained to sotorasib use in the following NSCLC clinical conditions: pancreatic carcinoma, product used for unknown indication, adenocarcinoma, colon cancer, colorectal cancer metastatic, colorectal cancer, pancreatic carcinoma stage IV, adenocarcinoma pancreas, pancreatic carcinoma metastatic, bile duct adenocarcinoma bile duct cancer, bronchial cancer metastatic, neoplasm malignant, KRAS gene mutation, targeted cancer therapy, unknown, colon cancer KRAS gene mutation, colangiocarcinoma, neoplasm malignant progression, esophageal carcinoma, rectal cancer metastatic, drug withdrawn, meningeal neoplasm, off-label use, bronchial cancer, and adenocarcinoma metastatic.

**Figure 1:**
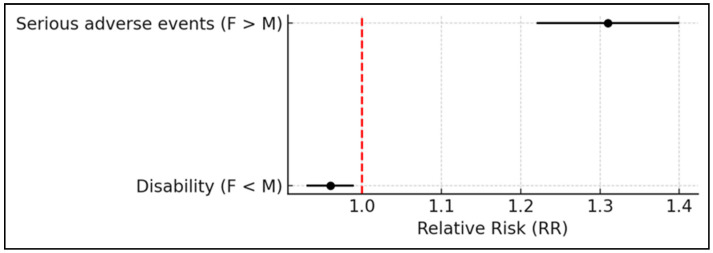
Forest plot representing the RR and corresponding 95% confidence intervals for sex-specific differences in adverse events. Female patients demonstrated a significantly increased risk of serious adverse events compared with males. In contrast, the risk of disability as an outcome was slightly reduced in females. The vertical red dashed line marks the null value (RR = 1.0), indicating no difference in risk between sexes.

Within the examined ICSRs, 49.2% pertained to male patients (26% in the 18–64 years age bracket, and 41% in the 65–85 years bracket), while 40.1% involved female patients (31% in the 18–64 years age bracket and 32% in the 65–85 years bracket). Among all ADRs, 47.5% were classified as serious, with 43.9% of these occurring in females. Notably, women exhibited a significantly higher risk of experiencing serious adverse events compared with men (RR: 1.31; 95% CI: 1.22–1.40; *p* < 0.0001) (Figure 1). The outcomes of ADRs were distributed between males and females as follows: hospitalization (26.5% vs. 32.7%), death (9.6% vs. 6.4%), disability (12% vs. 2% here “disability” refers to the SAE criterion defined by pharmacovigilance regulations, indicating a substantial disruption of a person’s ability to conduct normal life functions), life-threatening events (2.4% vs. 2.4%), and other medically important conditions (49.5% vs. 56.5%). Interestingly, the risk of disability as an outcome was slightly lower in females compared with males (RR = 0.96; 95% CI: 0.93–0.99; *p* = 0.01) (Figure 1).

**Figure 2:**
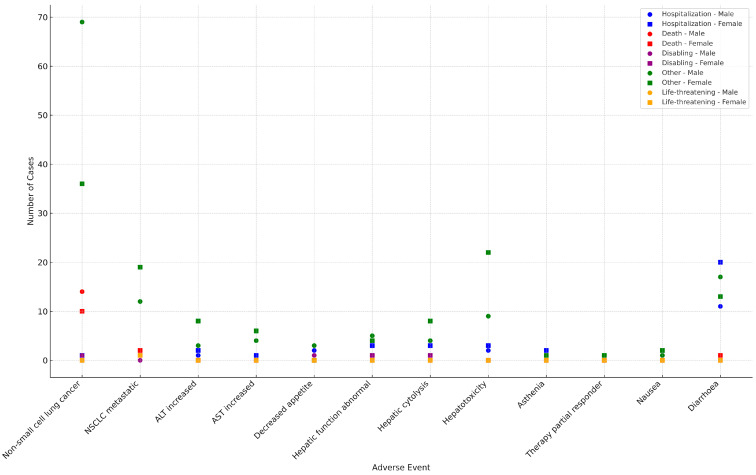
Distribution of the most frequently reported adverse event outcomes by sex in patients treated with sotorasib. The scatter plot shows the distribution of cases concerning the most commonly documented adverse events (AEs) in sotorasib-treated males and females. Outcomes of interest were hospitalization, death, disability, life-threatening events, and other significant medical conditions. Each point depicts the count for cases stratified by sex for a specific AE and outcome combination. Circles represent male cases and squares represent female cases, with color indicating the type of outcome.

The distribution of the most frequently reported adverse events outcomes by sex in patients treated with sotorasib is shown in Figure 2.

### 2.2. System Organ Classes Analysis/Assessment

A total of 24 system organ classes were implicated. Among the five most frequently affected were benign, malignant, and unspecified neoplasms (including cysts and polyps), accounting for 15.8% of the reports; gastrointestinal disorders (15.3%); investigations (15.1%); general disorders and administration site conditions (12.1%); and hepatobiliary disorders (11.5%). These data are presented in Table 1, where all SOCs are stratified by sex.

**Figure 3:**
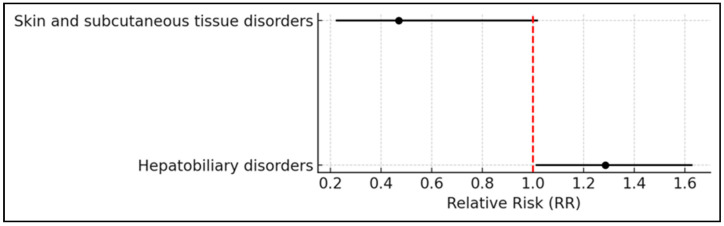
Forest plot showing the RR and 95% confidence intervals for adverse events with statistically significant sex-based differences (*p* < 0.05). The vertical red dashed line indicates the null value (RR = 1), representing no difference in risk between females and males. Events to the right of the line are more frequent in females; those to the left are more frequent in males.

Only skin and subcutaneous tissue disorders (46 reports) and hepatobiliary disorders (232 reports) met the criteria for a positive signal according to the disproportionality analysis. For hepatobiliary disorders, the RR was 1.29 with a 95% CI of 1.01 to 1.63, and the association was statistically significant (*p* < 0.05), with a higher risk observed in females. In contrast, skin and subcutaneous tissue disorders showed an RR of 0.47 (95% CI: 0.22 to 1.02), also statistically significant (*p* < 0.05), with a higher risk observed in males (Figure 3).

### 2.3. Preferred Terms Analysis/Assessment

**Figure 4:**
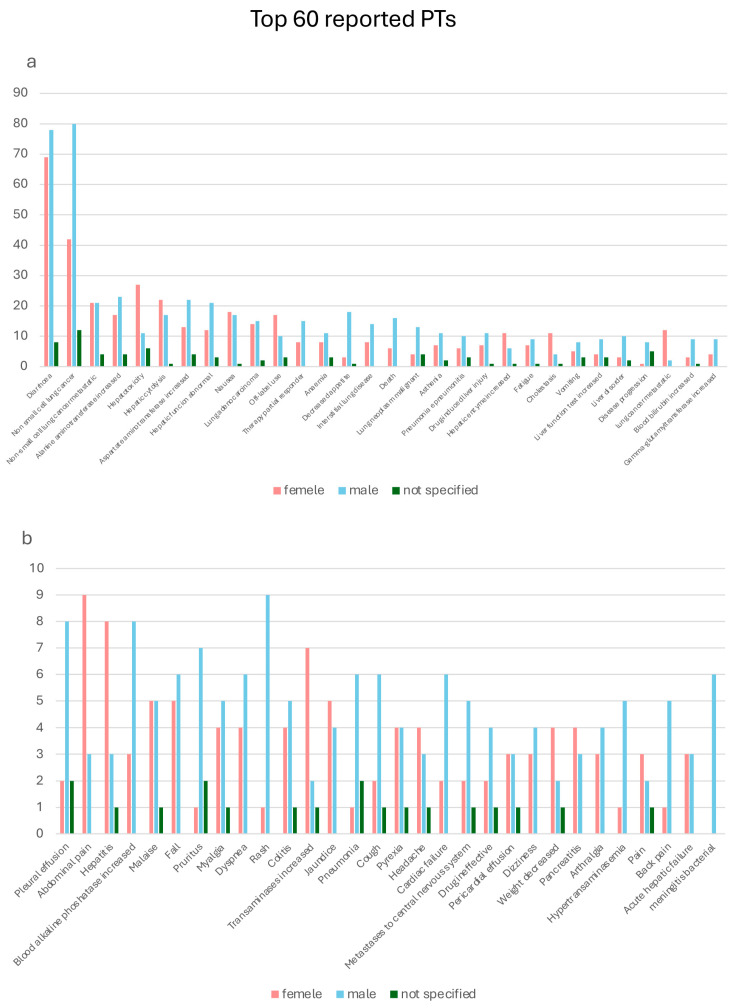
Top 60 preferred terms stratified by sex. (**a**) First 30 most common PTs; (**b**) PTs ranked from 31 to 60. Bar chart of the 60 most frequently reported PTs by sex. The most common events were diarrhea and non-small cell lung cancer (both primary and metastatic), followed by hepatotoxicity-related terms. A higher frequency of gastrointestinal and liver-related adverse events was observed in females, while some respiratory events (e.g., pneumonia, dyspnea) appeared more common in males. This visualization highlights sex-related differences in reporting patterns.

A total of 456 adverse events were collected, among which diarrhea (*n* = 155), non-small cell lung cancer (*n* = 134), metastatic non-small cell lung cancer (*n* = 46), increased alanine aminotransferase (*n* = 44), hepatotoxicity (*n* = 44), hepatic cytolysis (*n* = 40), increased aspartate aminotransferase (*n* = 39), abnormal hepatic function (*n* = 36), nausea (*n* 36), lung adenocarcinoma (*n* = 31), off-label (*n* = 30), partial therapy responder (*n* = 23), anemia (*n* = 22), decreased appetite (*n* = 22), interstitial lung disease (*n* = 22), death (*n* = 22), malignant lung neoplasm (*n* = 21), asthenia (*n* = 20), pneumonia and pneumonitis (*n* = 19), drug-induced liver injury (*n* = 19), increased hepatic enzyme (*n* = 18), fatigue (*n* = 17), cholestasis (*n* = 16), and vomiting (*n* = 16) were the 24 most reported. Figure 4 shows the top 60 preferred terms stratified by sex.

**Figure 5:**
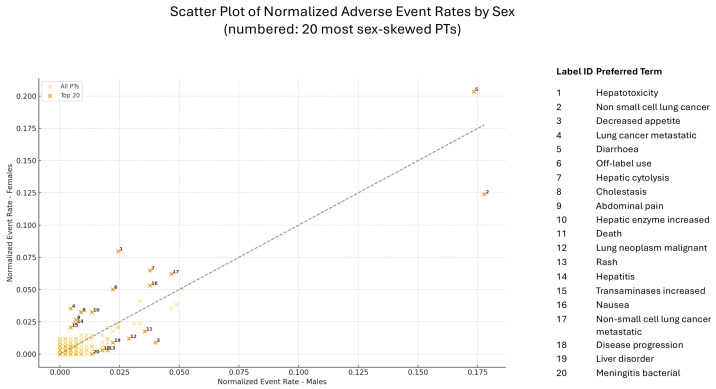
Scatter plot of normalized adverse event rates by sex. Each point corresponds to a PT; the 20 most sex-disparate PTs are numbered and listed in the legend. The dashed diagonal indicates parity between male and female event rates. Points above the line represent higher reporting rates in females, while those below indicate higher reporting rates in males. Key finding: hepatotoxicity and hepatic cytolysis were more frequently reported in females. Notably, a sex-specific pattern emerged for several adverse events. A total of four positive signals were identified. Females were at significantly greater risk for cholestasis (RR = 3.37; 95% CI: 1.08–10.50; *p* = 0.03) and hepatotoxicity (RR = 3.01; 95% CI: 1.52–5.98; *p* = 0.001), indicating a marked susceptibility to hepatobiliary toxicity. In contrast, males showed a greater probability of reporting of decreased appetite (RR = 7.35; 95% CI: 1.67–45.7; *p* = 0.0011) rash (RR = 7.3; 95% CI: 0.97–154.9; *p* = 0.02), and bacterial meningitis (RR = 10.6; 95% CI: 0.60–187.5; *p* = 0.036) compared with females (Figure 6).

A scatter plot of normalized adverse event rates stratified by sex, with male rates on the x-axis and female rates on the y-axis is presented in Figure 5. A clear asymmetry can be observed across several events, underscoring potential sex-based differences in drug response or disease manifestation. Among the most skewed PTs, hepatotoxicity demonstrated a notable elevation in female-normalized event rates, suggesting a disproportionate burden in women. Conversely, the PT “non-small cellular lung cancer” showed substantially higher normalized rates in males. Additional events such as decreased appetite, hepatic cytolysis, off-label use, and death also exhibited distinct sex-specific trends, either favoring one sex or showing marked divergence from the diagonal line.

**Figure 6:**
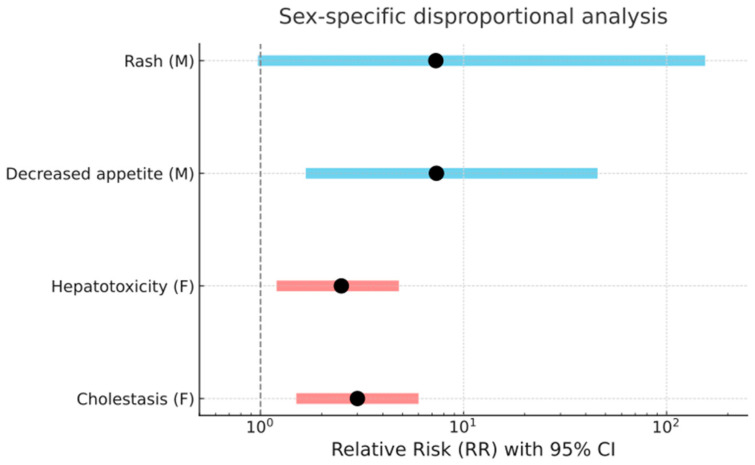
Sex-specific disproportional analysis for selected adverse events. Risk ratios are plotted on the x-axis, with each event labeled on the y-axis. The vertical dashed line at RR = 1 indicates no difference in risk between sexes. Bars are color-coded: pink indicates significantly higher risk in females, and light blue indicates higher risk in males. The black dots represent point estimates of RR. Events such as cholestasis and hepatotoxicity showed elevated risk in females, while decreased appetite and rash were more frequent in males.

### 2.4. Comparative Assessment of Safety Signals from RCTs and EV

A comparative analysis of adverse event rates between the literature-based cohort from CodeBreaK200 RCT (*n* = 345) and the real-world cohort (*n* = 845) revealed statistically significant differences across all six examined PT. Diarrhea was reported in 33.9% of patients in the CodeBreaK200 cohort versus 17.4% in the real-world cohort (z = 6.22, *p* < 0.0001), while fatigue occurred in 7.0% vs. 1.9% of patients, respectively (z = 4.40, *p* < 0.0001). Similarly, nausea was significantly more frequent in the CodeBreaK200 group (13.9%) compared with the real-world group (4.1%) (z = 6.00, *p* < 0.0001). Liver enzyme alterations, including increased alanine and aspartate transaminase level, also demonstrated significant discrepancies: alanine decrease was observed in 11.0% of the literature patients versus 4.7% in the real-world data (z = 3.97, *p* = 0.0001), and aspartate increase occurred in 11.0% vs. 4.1%, respectively (z = 4.48, *p* < 0.0001). Finally, decreased appetite was reported in 11.0% of the literature cohort and in 2.5% of the real-world group (z = 6.15, *p* < 0.0001).

### 2.5. Assessment of Onset Time of ADRs

An analysis of the onset time of toxicities was performed. In our cohort, the evaluation of toxicity onset revealed heterogeneous trends according to sex-related differences. Regarding PTs associated with the SOC investigations such as transaminase elevations, women experienced an earlier onset compared with men: mean of 50 days for ALT (SD 31.2; 95% CI: 27.6–127.6) and 45 days for AST (SD 42.4; 95% CI: 336.2–426.2) versus 72.3 days (SD 116.4; 95% CI: 49.8–194.4) and 105.8 days (SD 134.5; 95% CI: 108.3–319.8), respectively, in men. For overall hepatotoxicity, the mean onset was later in women (90.7 days, SD 66.0; 95% CI 21.4–159.9) compared with men (27 days, SD 16.6; 95% CI: 14.3–68.3). A similar pattern was observed for cholestasis, with a mean latency of 39.3 days in women (SD 23.5; 95% CI: 14.7–64.0) and 110 days in men (SD 45.3; 95% CI: 296.6–516.6). Among the non-hepatobiliary toxicities, nausea occurred at a mean of 55.7 days in women (SD 30.9; 95% CI 23.2–88.1) whereas it appeared earlier in men (5.7 days, SD 2.5; 95% CI: 0.6–11.9). Fatigue was reported exclusively in female patients (mean 51 days, SD 47.1; 95% CI: 66.1–168.1). Appetite loss showed high variability, with a single female case reported at 102 days, and a mean onset of 24 days in men (SD 31.1; 95% CI: 255.5–303.5). Rash and other cutaneous toxicities were rare, with insufficient data for a robust estimate.

## 3. Discussion

The emergence of targeted therapies, particularly those addressing the KRAS G12C mutation, marks a significant advance in the therapeutic landscape for NSCLC. In order to evaluate the sotorasib safety profile, the Phase 1/2 CodeBreaK 100 clinical trial was initiated in 2018, first enrolling patients with KRAS p.G12C-mutated solid tumors, and subsequently focusing on those with KRAS p.G12C-mutated NSCLC.

The Phase 1 portion was an open-label, multicenter study designed to assess dose safety and tolerability. A total of 129 patients were enrolled in dose-escalation and dose-expansion cohorts including 59 with NSCLC, 42 with colorectal cancer, and 28 with other tumor types. Treatment-related AEs (TRAEs) of any grade occurred in 73 patients (56.6%), with 11.6% experiencing serious treatment-related AEs. Grade 3 or 4 CTCAE (Common Terminology Criteria for Adverse Events) TRAEs occurred in 15 patients (11.6%). Specifically, Grade 3 TRAEs included alanine aminotransferase (ALT) increase (4.7%), diarrhea (3.9%), anemia (3.1%), aspartate aminotransferase (AST) increase (2.3%), blood alkaline phosphatase increase (1.6%), hepatitis (0.8%), lymphocyte count decrease (0.8%), gamma-glutamyl transferase increase (0.8%), and hyponatremia (0.8%). A single case of Grade 4 ALT increase was observed that resolved following dose reduction in sotorasib and the introduction of glucocorticoid tapering. In addition, one patient (0.8%) discontinued treatment due to Grade 3 elevations in ALT and AST levels associated with sotorasib (Table 2) [25].

The Phase 2 CodeBreaK100 trial evaluated the clinical efficacy and safety of sotorasib in 126 patients with locally advanced or metastatic NSCLC KRASG12C who had experienced disease progression following immunotherapy, platinum-based combination chemotherapy, or both. TRAEs of any grade were documented in 88 patients (69.8%), with Grade 3 TRAEs occurring in 25 patients (19.8%) and one patient (0.8%) experiencing a Grade 4 TRAE, specifically pneumonitis and dyspnea. No treatment-related Grade 5 events were reported. The most frequently observed TRAEs included diarrhea (31.7%), nausea (19.0%), alanine aminotransferase increase (15.1%), aspartate aminotransferase increase (15.1%), and fatigue (11.1%). The most common TRAEs necessitating dose adjustments were diarrhea (7.9%), aspartate aminotransferase increase (7.9%), alanine aminotransferase increase (7.1%), blood alkaline phosphatase increase (2.4%), and nausea (2.4%) (Table 3) [23].

Based on the favorable results of the CodeBreaK 100 trial, sotorasib received accelerated approval from the FDA on 28 May 2021 for the treatment of locally advanced or metastatic KRASG12C NSCLC [26].

CodeBreaK200 represents the first global, randomized, Phase 3 controlled trial designed to compare the efficacy, safety, and patient-reported outcomes of sotorasib versus standard-of-care docetaxel in patients with previously treated, advanced NSCLC KRASG12C mutation. A total of 345 patients with advanced KRASG12C-mutant NSCLC, who had received prior anticancer therapies, were enrolled and randomized in a 1:1 ratio to receive either oral sotorasib at a dose of 960 mg once daily or intravenous docetaxel at 75 mg/m^2^ every three weeks. TRAEs of any grade occurred in 98% of patients in both treatment arms. In the sotorasib group, the most frequent grade ≥3 TRAEs included diarrhea (12%), elevated alanine aminotransferase (8%), and elevated aspartate aminotransferase (5%) (Table 4). All grade ≥3 events related to diarrhea or elevated liver enzymes resolved following dose modification (interruption and/or reduction). One fatal TRAE (interstitial lung disease) was reported in the sotorasib arm (<1%). A post hoc analysis revealed a higher incidence of grade ≥3 TRAEs and hepatotoxicity in patients who had received immunotherapy within 2.6 months prior to initiating sotorasib compared with those whose immunotherapy occurred more than 2.6 months before. Overall, a longer interval between prior immunotherapy and sotorasib initiation was associated with a reduced incidence of high-grade treatment-related toxicity, particularly hepatotoxicity. The most frequent treatment-related AEs leading to sotorasib discontinuation were hepatotoxicity events, which were more prevalent when prior immunotherapy had been administered within a short interval before sotorasib treatment—consistent with observations from the expanded-access program. The Phase 3 CodeBreaK 200 trial showed a significant improvement in progression-free survival with sotorasib (median progression free survival (PFS): 5.6 vs. 4.5 months; hazard ratio (HR): 0.66; *p* = 0.0017) and a higher overall response rate (28.1% vs. 13.2%). However, there was no significant difference in overall survival (OS) between the two groups (median OS: 10.6 months for sotorasib vs. 11.3 months for docetaxel) [27]. These findings establish sotorasib as the first oral targeted agent in a Phase 3 randomized setting to significantly improve both the PFS and overall response rate over standard chemotherapy in this patient population [27].

This study aimed to evaluate the safety of the EMA-approved therapy sotorasib by integrating real-world data from EV with evidence from RCTs. Sex and age distribution revealed a slight male predominance, although serious ADRs were disproportionately higher in females.

Multiple international analyses have demonstrated that females exhibit a 1.5- to 2-fold increased risk of developing adverse drug reactions (ADRs) relative to men, and consequently experience higher rates of ADR-related hospitalization [28]. This risk partly reflects the underrepresentation of women in early phase clinical trials, leading to drug dosages derived from predominantly male populations that may not be generalizable to women [29]. Both biological sex and gender-related factors contribute to this discrepancy. Biological differences include variations in pharmacogenomics, pharmacokinetics, pharmacodynamics, gut microbiota composition, hormone exposure (endogenous and exogenous), and sex-specific pharmacogenetics such as cytochrome P450 isoforms (notably CYP3A, with higher activity in women). Gender-related aspects include higher rates of polypharmacy among women, leading to more drug–drug interactions as well as differences in the reporting of ADRs, with women tending to report more frequently [30]. Women generally experience more severe symptomatic and hematological adverse events across various treatment modalities. In the context of immunotherapy, sex-related differences are particularly evident: female patients with metastatic melanoma or non-small cell lung cancer (NSCLC) appear to face a higher risk of immune-related adverse events than their male counterparts. Endocrine toxicities also demonstrate sex specificity, with thyroid dysfunction more frequent in women and pituitary toxicity more common in men. These findings underscore the importance of evaluating immunotherapy-related adverse events through a sex- and gender-informed perspective [31,32].

Consistent with previous reports, our study observed a slight male predominance in the cohort, while ADRs were disproportionately higher among female patients.

In particular, women had a 33% higher risk of experiencing serious ADRs than men, while men had a 4.2% higher risk than women of reporting disability as an outcome. The disproportionality analysis identified four sex-specific safety signals. Women had a threefold higher risk of developing cholestasis and a threefold higher risk of hepatotoxicity compared with men. Conversely, men were significantly more likely to experience decreased appetite and rash. This finding aligns with previous reports [23,25,27] indicating sex-based differences in liver metabolism and drug clearance and suggests potential sex-based biological susceptibilities or disparities in drug safety profile, warranting further investigation. In line with these results, previous studies have emphasized the role of sex in drug-induced liver injury (DILI) and cholestasis. In particular, Ismail et al. [33] demonstrated sex-dependent differences in cholestasis, highlighting the higher susceptibility of females to bile flow impairment and related hepatobiliary complications due to the influence of estrogen. Similarly, Amacher (2014) [34] identified female sex as a susceptibility factor for DILI, potentially driven by differences in hepatic enzyme expression and drug metabolism. Supporting these observations, in our analysis, the SOC *Hepatobiliary disorders* was disproportionately represented in women. A further aspect that emerged from our analysis concerns the timing of the onset of toxicities associated with sotorasib in real-world clinical practice. As reported in the literature, some adverse events, particularly those involving the hepatobiliary system [35], tend to occur in the early stages of clinical trials, supporting the recommendation of intensive laboratory monitoring during the first months of treatment, especially in patients who have received immunotherapy in the 2–6 months prior to starting sotorasib treatment. In our dataset, which was extremely limited, the analysis of onset times revealed potential gender-related differences, with earlier increases in transaminases in women, while hepatotoxicity and cholestasis appeared later than in men. However, the wide confidence intervals and observed variability highlight a high degree of statistical uncertainty, likely due to the limited sample size and incomplete availability of onset data. These findings should therefore be considered exploratory. Extending the comparative analysis between the literature data from registered trials and real-world ADR patterns, significant discrepancies emerged. Adverse events such as diarrhea, fatigue, nausea, and transaminase elevations were consistently more frequent in the RCT cohort. These differences may reflect the rigorous monitoring and standardized reporting inherent to clinical trials, which often capture milder or transient events that might be underreported in routine care settings. To further illustrate these discrepancies, we compared the most frequently reported TRAEs across different phases of the CodeBreaK program and real-world pharmacovigilance data (Table 5). The analysis confirmed a consistent overrepresentation of gastrointestinal (e.g., diarrhea, nausea, vomiting) and hepatic (e.g., transaminase elevations) toxicities in RCTs compared with real-world reports. For example, diarrhea was observed in up to 34% of patients in CodeBreaK 200, whereas only 8.2% of cases were reported in the EV database. Similarly, ALT and AST increases were reported in 10–15% of patients in clinical trials but accounted for approximately 2% in the EV dataset. Conversely, unexpected toxicities such as hepatitis or hyponatremia were captured in real-world data, reflecting the complementary value of spontaneous reporting systems in detecting signals that may not emerge in controlled trial settings. The proportional z-test confirmed statistically significant divergence in all six key adverse events, underscoring the value of post-marketing surveillance in complementing trial-based safety profiles.

Taken together, these findings underscore that both sex and gender play crucial roles in drug safety, efficacy, and outcomes in oncology. Increasing female representation in clinical trials and designing studies that enable meaningful sex-based subgroup analyses are essential steps toward more precise and equitable cancer treatment.

### Strengths and Limitations

Spontaneous reporting system (SRS) analyses remain among the most widely utilized and effective pharmacovigilance approaches for generating potential safety signals that merit further investigation and validation [36]. With the increasing clinical use of sotorasib and other covalent inhibitors, including adagrasib, a comprehensive assessment of the safety profiles of these drugs is needed, particularly in light of emerging evidence suggesting that sex-related biological differences may influence both the incidence and severity of ADRs. Sex has widely been demonstrated to affect the response to immunotherapeutic agents in patients with several solid tumors, including NSCLC, probably reflecting differences in immune profiles as well as in genetic, hormonal, environmental, and microbiome composition between men and women. These biological differences can potentially affect responses and toxicity to targeted agents [37].

Understanding these sex-specific safety variations is essential to optimize personalized treatment strategies, minimize the risk of adverse reactions, and improve cancer patient management. The main strength of this study lies in its contribution to the growing knowledge on sex-specific safety outcomes through the use of the EV database and employing disproportional analysis as a statistical method. Nonetheless, spontaneous reports are subject to limitations, including incomplete or inconsistent data, and may lack crucial clinical information such as patient clinical characteristics, comorbidities, concurrent therapies and outcomes as well as molecular data [38]. There may also be a bias toward the underreporting of known or expected adverse effects such as nausea, vomiting, and fatigue. Moreover, targeted therapies are generally associated with a lower incidence and severity of ADRs. Importantly, our analysis should be considered as a signal-generation study, aimed at highlighting potential sex-related differences in safety outcomes. Further investigations including prospective and mechanistic studies will be required as more clinical data become available to validate and expand upon these preliminary observations.

This could allow for a more personalized approach to KRAS-mutant patients not only to improve the efficacy, but also to spare toxicity and preserve the quality of life.

## 4. Materials and Methods

This study analyzed data from the EV database, accessed via https://www.adrreports.eu/it/index.html on 8 April 2025. Sotorasib related ICSRs were collected from 1 January 2021 to 8 April 2025 and reports with other therapeutic indications were excluded. Inclusion criteria consisted of all reports in which sotorasib was used in patients with KRAS-mutated NSCLC. Reports with other therapeutic indications were excluded. The selection process was conducted at the case level using the individual case identification number. ADRs were classified by using the Medical Dictionary for Regulatory Activities (MedDRA)^®^ 26.0 dictionary. Data were analyzed by using both descriptive and disproportionality methods. Descriptive analyses included stratification by age, seriousness ADRs, and PTs. Disproportionality was assessed through RRs with corresponding 95% CIs to assess the differences between sex. In analyses where a cell frequency of zero occurred, the Haldane–Anscombe correction was applied to avoid bias in the estimation of the RR and CI.

To reduce the likelihood of highlighting spurious associations, only PTs with statistically significant disproportionality (*p* < 0.05) were considered in the comparative discussion.

In addition, a brief review of the available literature on the safety profile of sotorasib was conducted, incorporating data from randomized clinical trials. In order to evaluate differences in ADR reporting between RCTs and the EV database, a two-proportion z-test was performed. This statistical method enabled a direct comparison of reporting frequencies across the two data sources, highlighting potential discrepancies between controlled trial settings and real-world pharmacovigilance data.

## 5. Conclusions

This pharmacovigilance analysis, integrating real-world data from EV with evidence from the CodeBreaK200 randomized clinical trial, revealed both concordances and notable discrepancies in the safety profile of sotorasib. Real-world data confirmed key adverse events identified in clinical trials but indicated lower overall reporting frequencies, likely reflecting differences in monitoring intensity and reporting practices. Importantly, the study highlighted marked sex-specific differences: women had an approximately threefold higher risk of developing cholestasis and hepatotoxicity while men were significantly more likely to experience decreased appetite and rash. These findings underscore the importance of incorporating sex as a biological variable in post-marketing safety assessments and suggest that tailored monitoring strategies may be warranted. Integrating trial data with real-world pharmacovigilance enhances the understanding of drug safety, supports risk minimization strategies, and contributes to more personalized therapeutic decision-making.

## Figures and Tables

**Table 1:** SOCs are stratified by sex.

SOC	Total (%)	Female (*n*)	Male (*n*)	Not Specified	RR
Neoplasms benign, malignant, and unspecified (incl cysts and polyps)	320 (15.8%)	116 (13.7%)	160 (18.9%)	44 (5.2%)	0.94
Gastrointestinal disorders	310 (15.3%)	141 (16.7%)	155 (18.3%)	14 (1.7%)	1.18
Investigations	306 (15.1%)	103 (12.2%)	153 (18.1%)	50 (5.9%)	0.87
General disorders and administration site conditions	244 (12.1%)	79 (9.3%)	122 (14.4%)	43 (5.1%)	0.84
Hepatobiliary disorders	232 (11.5%)	115 (13.6%)	116 (13.7%)	1 (0.1%)	1.29
Respiratory, thoracic, and mediastinal disorders	148 (7.3%)	40 (4.7%)	74 (8.8%)	34 (4.0%)	0.70
Nervous system disorders	86 (4.3%)	23 (2.7%)	43 (5.1%)	20 (2.4%)	0.69
Injury, poisoning, and procedural complications	56 (2.8%)	29 (3.4%)	22 (2.6%)	5 (0.6%)	1.71
Cardiac disorders	46 (2.3%)	16 (1.9%)	23 (2.7%)	7 (0.8%)	0.90
Skin and subcutaneous tissue disorders	46 (2.3%)	8 (0.9%)	23 (2.7%)	15 (1.8%)	0.07
Blood and lymphatic system disorders	44 (2.2%)	20 (2.4%)	22 (2.6%)	2 (0.2%)	1.18
Musculoskeletal and connective tissue disorders	42 (2.1%)	19 (2.2%)	21 (2.5%)	2 (0.2%)	1.17
Infections and infestations	42 (2.1%)	18 (2.1%)	21 (2.5%)	3 (0.4%)	1.11
Vascular disorders	26 (1.3%)	5 (0.6%)	13 (1.5%)	8 (0.9%)	0.50
Renal and urinary disorders	23 (1.1%)	13 (1.5%)	10 (1.2%)	0 (0%)	1.69
Psychiatric disorders	11 (0.5%)	8 (0.9%)	3 (0.4%)	0 (0%)	3.46
Ear and labyrinth disorders	10 (0.5%)	4 (0.5%)	5 (0.6%)	1 (0.1%)	1.04
Surgical and medical procedures	10 (0.5%)	3 (0.4%)	5 (0.6%)	2 (0.2%)	0.78
Eye disorders	5 (0.2%)	4 (0.5%)	1 (0.1%)	0 (0%)	5.19
Immune system disorders	5 (0.2%)	3 (0.4%)	2 (0.2%)	0 (0%)	1.95
Metabolism and nutrition disorders	4 (0.2%)	2 (0.2%)	2 (0.2%)	0 (0%)	1.10
Congenital familiar and genetic disorders	4 (0.2%)	0 (0%)	2 (0.2%)	2 (0.2%)	-
Endocrine disorders	2 (0.1%)	1 (0.1%)	1 (0.1%)	0 (0%)	1.04
Reproductive system and breast disorders	1 (0.1%)	1 (0.1%)	0 (0%)	0 (0%)	1.23

Note: SOC, system organ class.

**Table 2:** Sotorasib safety profile from the Phase 1 CodeBreaK 100 trial.

TRAE	Grade 3	Grade 4
Alanine aminotransferase increase	4.7%	0.8%
Diarrhea	3.9%	
Anemia	3.1%	
Aspartate aminotransferase increase	2.3%	
Blood alkaline phosphatase increase	1.6%	
Hepatitis	0.8%	
Decreased lymphocyte count	0.8%	
Gamma-glutamyl transferase increase	0.8%	
Hyponatremia	0.8%	

Note: TRAE, treatment-related adverse event.

**Table 3:** Sotorasib safety profile from the Phase 2 CodeBreaK 100 trial.

TRAE	Any Grade	Grade ≥ 3
Diarrhea	31.7%	4.0%
Nausea	19%	0.0%
Alanine aminotransferase increase	15.1%	6.3%
Aspartate aminotransferase increase	15.1%	5.6%
Fatigue	11.1%	0.0%
Vomiting	7.9%	0.0%
Gamma-glutamyl transferase increase	2.4%	2.4%
Hepatotoxic event	0.8%	0.8%
Dyspnea	1.6%	0.8%

Note: TRAE, treatment-related adverse event.

**Table 4:** Sotorasib safety profile from the CodeBreaK 200 clinical trial.

TRAE	Any Grade	Grade ≥ 3
Diarrhea	34%	12%
Fatigue	7%	1%
Nausea	14%	1%
Decreased appetite	11%	2%
Alanine aminotransferase increased	10%	8%
Aspartate aminotransferase increased	10%	5%

Note: TRAE, treatment-related adverse event.

**Table 5:** Differences in the incidence of adverse events between the clinical trial reports (CodeBreaK program) and real-world data. NA: not applicable.

TRAE	Phase 1 CodeBreaK 100 Trial	Phase 2 CodeBreaK 100 Trial	CodeBreaK 200 Clinical Trial	Real-World Evidence
Alanine aminotransferase increase	11.6%	15.1%	10%	2.3%
Anemia	13.2%	NA	3%	1.2%
Aspartate aminotransferase increase	13.2%	15.1%	10%	2.1%
Blood alkaline phosphatase increase	NA	NA	7%	0.6%
Decreased appetite	14.7%	NA	11%	1.2%
Decreased lymphocyte count	NA	2.4%	NA	0.1%
Diarrhea	29.5%	31.7%	34%	8.2%
Dyspnea	16.3%	1.6%	NA	0.5%
Fatigue	23.3%	11.1%	7%	0.9%
Gamma-glutamyl transferase increase	NA	2.4%	NA	0.7%
Hepatitis	NA	NA	NA	0.6%
Hepatotoxic event	NA	0.8%	NA	2.3%
Hyponatremia	NA	NA	NA	0.1%
Nausea	20.9%	19%	14%	1.9%
Vomiting	17.8%	7.9%	5%	0.9%

## Data Availability

The original contributions presented in this study are included in the article. Further inquiries can be directed to the corresponding author.

## Abbreviations

The following abbreviations are used in this manuscript:

ADR	Adverse drug reaction
AE	Adverse event
ALK	Anaplastic lymphoma kinase
ALT	Alanine aminotransferase
AST	Aspartate aminotransferase
CI	Confidence interval
CTCAE	Common Terminology Criteria for Adverse Events
DILI	Drug-induced liver injury
EGFR	Epidermal growth factor receptor
EV	EudraVigilance
FDA	Food and Drug Administration
GTP	Guanosine triphosphate
HR	Hazard ratio
ICSRS	Individual case safety reports
KEAP1	Kelch-like ECH-associated protein 1
KRAS	Kirsten rat sarcoma
LKB1	Liver kinase B1
MedDRA	Medical Dictionary for Regulatory Activities
NGS	Next-generation sequencing
NSCLC	Non-small cell lung cancer
NTRK	Neurotrophic tyrosine receptor kinase
PDGFRA	Platelet-derived growth factor receptor alpha
PD-L1	Programmed death-ligand 1
PFS	Progression free survival
PT	Preferred term
PTEN	Phosphatase and tensin homolog
RR	Relative risk
RTC	Randomized clinical trial
SOC	System organ class
SRS	Spontaneous reporting system
STK11	Serine/threonine kinase 11
TRAE	Treatment-related AE

## References

[1] Bray F., Laversanne M., Sung H., Ferlay J., Soerjomataram I., Siegel R.L., Torre L.A., Jemal A. (2024). Global Cancer Statistics 2022: GLOBOCAN Estimates of Incidence and Mortality Worldwide for 36 Cancers in 185 Countries. CA Cancer J. Clin..

[2] Huang Q., Li Y., Huang Y., Wu J., Bao W., Xue C., Li X., Dong S., Dong Z., Hu S. (2025). Advances in Molecular Pathology and Therapy of Non-Small Cell Lung Cancer. Signal Transduct. Target Ther..

[3] Hendriks L.E.L., Cortiula F., Martins-Branco D., Mariamidze E., Popat S., Reck M., ESMO Guidelines Committee (2025). Updated Treatment Recommendations for Systemic Treatment: From the ESMO Oncogene-Addicted Metastatic NSCLC Living Guideline. Ann. Oncol..

[4] Odintsov I., Sholl L.M. (2024). Prognostic and Predictive Biomarkers in Non-Small Cell Lung Carcinoma. Pathology.

[5] Cancer Genome Atlas Research Network (2014). Comprehensive Molecular Profiling of Lung Adenocarcinoma. Nature.

[6] Judd J., Abdel Karim N., Khan H., Naqash A.R., Baca Y., Xiu J., VanderWalde A.M., Mamdani H., Raez L.E., Nagasaka M. (2021). Characterization of KRAS Mutation Subtypes in Non-Small Cell Lung Cancer. Mol. Cancer Ther..

[7] Prior I.A., Hood F.E., Hartley J.L. (2020). The Frequency of Ras Mutations in Cancer. Cancer Res..

[8] Riely G.J., Kris M.G., Rosenbaum D., Marks J., Li A., Chitale D.A., Nafa K., Riedel E.R., Hsu M., Pao W. (2008). Frequency and Distinctive Spectrum of KRAS Mutations in Never Smokers with Lung Adenocarcinoma. Clin. Cancer Res..

[9] Santarpia M., Ciappina G., Spagnolo C.C., Squeri A., Passalacqua M.I., Aguilar A., Gonzalez-Cao M., Giovannetti E., Silvestris N., Rosell R. (2023). Targeted Therapies for KRAS-Mutant Non-Small Cell Lung Cancer: From Preclinical Studies to Clinical Development—A Narrative Review. Transl. Lung Cancer Res..

[10] Rosell R., Codony-Servat J., González J., Santarpia M., Jain A., Shivamallu C., Wang Y., Giménez-Capitán A., Molina-Vila M.A., Nilsson J. (2024). KRAS G12C-Mutant Driven Non-Small Cell Lung Cancer (NSCLC). Crit. Rev. Oncol. Hematol..

[11] Soh J., Toyooka S., Matsuo K., Yamamoto H., Wistuba I.I., Lam S., Fong K.M., Gazdar A.F., Miyoshi S. (2015). Ethnicity Affects EGFR and KRAS Gene Alterations of Lung Adenocarcinoma. Oncol. Lett..

[12] Loong H.H., Du N., Cheng C., Lin H., Guo J., Lin G., Li M., Jiang T., Shi Z., Cui Y. (2020). KRAS G12C Mutations in Asia: A Landscape Analysis of 11,951 Chinese Tumor Samples. Transl. Lung Cancer Res..

[13] Wagner P.L., Stiedl A.C., Wilbertz T., Petersen K., Scheble V., Menon R., Reischl M., Mikut R., Rubin M.A., Fend F. (2011). Frequency and Clinicopathologic Correlates of KRAS Amplification in Non-Small Cell Lung Carcinoma. Lung Cancer.

[14] Sun J.M., Hwang D.W., Ahn J.S., Ahn M.J., Park K. (2013). Prognostic and Predictive Value of KRAS Mutations in Advanced Non-Small Cell Lung Cancer. PLoS ONE.

[15] Sholl L.M., Cooper W.A., Kerr K.M., Tan D.S.W., Tsao M.-S., Yang J.C.-H. (2023). IASLC Atlas of Molecular Testing for Targeted Therapy in Lung Cancer.

[16] Arbour K.C., Jordan E., Kim H.R., Dienstag J., Yu H.A., Sanchez-Vega F., Lito P., Berger M., Solit D.B., Hellmann M. (2018). Effects of Co-Occurring Genomic Alterations on Outcomes in Patients with KRAS-Mutant Non-Small Cell Lung Cancer. Clin. Cancer Res..

[17] Vaclova T., Chakraborty A., Sherwood J., Ross S., Carroll D., Barrett J.C., Downward J., de Bruin E.C. (2022). Concomitant KRAS Mutations Attenuate Sensitivity of Non-Small Cell Lung Cancer Cells to KRAS G12C Inhibition. Sci. Rep..

[18] Li D., Zhu X., Wang H., Li N. (2017). Association between PD-L1 Expression and Driven Gene Status in NSCLC: A Meta-Analysis. Eur. J. Surg. Oncol..

[19] Finn S.P., Addeo A., Dafni U., Thunnissen E., Bubendorf L., Madsen L.B., Biernat W., Verbeken E., Hernandez-Losa J., Marchetti A. (2021). Prognostic Impact of KRAS G12C Mutation in Patients with NSCLC: Results from the European Thoracic Oncology Platform Lungscape Project. J. Thorac. Oncol..

[20] Sebastian M., Eberhardt W.E.E., Hoffknecht P., Metzenmacher M., Wehler T., Kokowski K., Alt J., Schütte W., Büttner R., Heukamp L.C. (2021). KRAS G12C-Mutated Advanced Non-Small Cell Lung Cancer: A Real-World Cohort from the German Prospective, Observational, Nationwide CRISP Registry (AIO-TRK-0315). Lung Cancer.

[21] Santos E., Martin-Zanca D., Reddy E.P., Pierotti M.A., Della Porta G., Barbacid M. (1984). Malignant Activation of a K-ras Oncogene in Lung Carcinoma but Not in Normal Tissue of the Same Patient. Science.

[22] Canon J., Rex K., Saiki A.Y., Mohr C., Cooke K., Bagal D., Gaida K., Holt T., Knutson C.G., Koppada N. (2019). The Clinical KRAS(G12C) Inhibitor AMG 510 Drives Anti-Tumour Immunity. Nature.

[23] Skoulidis F., Li B.T., Dy G.K., Price T.J., Falchook G.S., Wolf J., Italiano A., Schuler M., Borghaei H., Barlesi F. (2021). Sotorasib for Lung Cancers with KRAS p.G12C Mutation. N. Engl. J. Med..

[24] Skoulidis F., Li B.T., de Langen A.J., Hong D.S., Lena H., Wolf J., Dy G.K., Curioni Fontecedro A., Tomasini P., Velcheti V. (2025). Molecular Determinants of Sotorasib Clinical Efficacy in KRASG12C-Mutated Non-Small-Cell Lung Cancer. Nat. Med..

[25] Hong D.S., Fakih M.G., Strickler J.H., Desai J., Durm G.A., Shapiro G.I., Falchook G.S., Price T.J., Sacher A., Denlinger C.S. (2020). KRASG12C Inhibition with Sotorasib in Advanced Solid Tumors. N. Engl. J. Med..

[26] Nakajima E.C., Drezner N., Li X., Mishra-Kalyani P.S., Liu Y., Zhao H., Bi Y., Liu J., Rahman A., Wearne E. (2022). FDA Approval Summary: Sotorasib for KRAS G12C-Mutated Metastatic NSCLC. Clin. Cancer Res..

[27] de Langen A.J., Johnson M.L., Mazieres J., Dingemans A.C., Mountzios G., Pless M., Wolf J., Schuler M., Lena H., Skoulidis F. (2023). Sotorasib versus Docetaxel for Previously Treated Non-Small-Cell Lung Cancer with KRASG12C Mutation: A Randomised, Open-Label, Phase 3 Trial. Lancet.

[28] Zucker I., Prendergast B.J. (2020). Sex differences in pharmacokinetics predict adverse drug reactions in women. Biol. Sex Differ..

[29] Jene K., Meyers D.E., Prasad V. (2021). The inclusion of women in global oncology drug trials over the past 20 years. JAMA Oncol..

[30] Özdemir B.C., Gerard C.L., Espinosa da Silva C. (2022). Sex and gender differences in anticancer treatment toxicity: A call for revisiting drug dosing in oncology. Endocrinology.

[31] Triggianese P., Novelli L., Galdiero M.R., Chimenti M.S., Conigliaro P., Perricone R., Perricone C., Gerli R. (2020). Immune checkpoint inhibitors-induced autoimmunity: The impact of gender. Autoimmun. Rev..

[32] Rubino R., Marini A., Roviello G., Presotto E.M., Desideri I., Ciardetti I., Brugia M., Pimpinelli N., Antonuzzo L., Mini E. (2021). Endocrine-related adverse events in a large series of cancer patients treated with anti-PD1 therapy. Endocrine.

[33] Ismail A., Kennedy L., Francis H. (2023). Sex-dependent differences in cholestasis: Why estrogen signaling may be a key pathophysiological driver. Am. J. Pathol..

[34] Amacher D.E. (2014). Female gender as a susceptibility factor for drug-induced liver injury. Hum. Exp. Toxicol..

[35] Desai A., Bai X., Lin J., Villegas A., Patel R., Gadgeel S., Leighl N., Sabari J., Liu S.V., Nagasaka M. (2023). Time from Immune Checkpoint Inhibitor to Sotorasib Use Correlates with Risk of Hepatotoxicity in Non-Small Cell Lung Cancer: A Brief Report. Cancer Treat. Res. Commun..

[36] Speranza D., Omero F., Cianci V., Marafioti M., Infurna C., Carroccio P., Spina E., Barbieri M.A., Esposito E., Silvestris N. (2025). Comparison Study of the Safety Profile of Olaparib Versus Niraparib: Analysis of Real-World Data from EudraVigilance. Pharmaceuticals.

[37] Conforti F., Pala L., Pagan E., Corti C., Bagnardi V., Queirolo P., Catania C., De Pas T., Giaccone G. (2021). Sex-based differences in response to anti-PD-1 or PD-L1 treatment in patients with non-small-cell lung cancer expressing high PD-L1 levels: A systematic review and meta-analysis of randomized clinical trials. ESMO Open.

[38] Salvador M.R., Monteiro C., Pereira L., Duarte A.P. (2022). Quality of spontaneous reports of adverse drug reactions sent to a regional pharmacovigilance unit. Int. J. Environ. Res. Public Health.

